# Lipoprotein-apheresis reduces circulating microparticles in individuals with familial hypercholesterolemia[S]

**DOI:** 10.1194/jlr.M049726

**Published:** 2014-10

**Authors:** Katherine D. Connolly, Gareth R. Willis, Dev B. N. Datta, Elizabeth A. Ellins, Kristin Ladell, David A. Price, Irina A. Guschina, D. Aled Rees, Philip E. James

**Affiliations:** * Institute of Molecular and Experimental Medicine School of Medicine, Cardiff University, Cardiff CF14 4XN, United Kingdom; ** Institute of Infection and Immunity, School of Medicine, Cardiff University, Cardiff CF14 4XN, United Kingdom; † Lipid Unit, Llandough Hospital, Cardiff CF64 2XX, United Kingdom; § Institute of Life Sciences, College of Medicine, Swansea University, Singleton Park, Swansea SA2 8PP, United Kingdom; †† School of Biosciences, Cardiff University, Cardiff CF10 3AX, United Kingdom

**Keywords:** extracellular vesicles, microvesicles, exosomes, low density lipoprotein-apheresis, phosphatidylserine, nanoparticle tracking analysis, tunable resistive pulse sensing, flow cytometry, fatty acids

## Abstract

Lipoprotein-apheresis (apheresis) removes LDL-cholesterol in patients with severe dyslipidemia. However, reduction is transient, indicating that the long-term cardiovascular benefits of apheresis may not solely be due to LDL removal. Microparticles (MPs) are submicron vesicles released from the plasma membrane of cells. MPs, particularly platelet-derived MPs, are increasingly being linked to the pathogenesis of many diseases. We aimed to characterize the effect of apheresis on MP size, concentration, cellular origin, and fatty acid concentration in individuals with familial hypercholesterolemia (FH). Plasma and MP samples were collected from 12 individuals with FH undergoing routine apheresis. Tunable resistive pulse sensing (np200) and nanoparticle tracking analysis measured a fall in MP concentration (33 and 15%, respectively; *P* < 0.05) pre- to post-apheresis. Flow cytometry showed MPs were predominantly annexin V positive and of platelet (CD41) origin both pre- (88.9%) and post-apheresis (88.4%). Fatty acid composition of MPs differed from that of plasma, though apheresis affected a similar profile of fatty acids in both compartments, as measured by GC-flame ionization detection. MP concentration was also shown to positively correlate with thrombin generation potential. In conclusion, we show apheresis nonselectively removes annexin V-positive platelet-derived MPs in individuals with FH. These MPs are potent inducers of coagulation and are elevated in CVD; this reduction in pathological MPs could relate to the long-term benefits of apheresis.

Familial hypercholesterolaemia (FH) is a common genetic disorder that causes elevated levels of atherogenic lipoproteins in the plasma, particularly LDL cholesterol. Over 85% of FH cases are caused by mutations in the LDL receptor rendering these receptors unable to bind or internalize LDL and leading to accumulation in the plasma (1). The disease follows an autosomal dominant pattern of inheritance and can result in heterozygote or more serious homozygote forms (2). In severe forms of FH, diet alteration and lipid lowering medications are often insufficient to lower LDL levels enough to abate atherosclerotic plaque formation (3). These patients therefore require frequent (bi-weekly) lipoprotein-apheresis (hereafter referred to as “apheresis”) treatments in combination with dietary and pharmacological intervention to control LDL levels (4).

Apheresis is a safe well-established procedure for the extracorporeal removal of LDL. Blood is removed from one arm and passed through a column to remove atherogenic lipoproteins before being returned to the body via the other arm. Different apheresis techniques may be utilized, but all reduce LDL by approximately 70% immediately following treatment (5–9). However, posttreatment LDL levels are not maintained, with levels rising to 50% of pretreatment values within 2–4 days (10). Despite this transiency, apheresis is associated with superior long-term cardiovascular benefits compared with alternative therapies (11–15).

Microparticles (MPs) are heterogeneous submicron vesicles released from many different cell types. Several terms exist to describe these vesicles, though here we use “MPs” as an umbrella term to encompass vesicles ranging from 30 nm to 1 μm in diameter. This range includes both exosomes and microvesicles. Exosomes are classically 30–100 nm in size and are produced from intracellular processing of endocytosed material, which is then incorporated into exosomes and released from the cell by exocytosis. Microvesicles are vesicles ranging from 100 nm to 1 μm released directly from the plasma membrane of cells in response to cellular activation or apoptosis. The phospholipid asymmetry of the cells’ plasma membrane is disrupted and, consequently, phosphatidylserine (PS) is externalized to the outer membrane. This is commonly used to identify microvesicle populations via annexin V binding. MPs are released both in vivo and in vitro into the external environment of many cell types, allowing subsequent isolation from biological fluids or conditioned culture media (16).

Increased numbers of MPs, particularly those derived from platelets, have been reported in many CVDs (17, 18), though their function in both health and disease remains poorly understood. The surface of platelet MPs is reportedly up to 100-fold more procoagulant than that of activated platelets due to an increased density of PS, P-selectin, and factor X (19). Furthermore, MPs are known to carry specific mRNAs, microRNAs, proteins, and lipid signaling molecules. These bioactive entities are thought to be delivered to target cells, though the mechanism by which this interaction occurs is still unknown (20). To date, little is known about the lipid concentration and profile of MPs, though platelet MPs have been shown to transfer pro-inflammatory lipids to platelets leading to activation (20).

Heterozygous FH patients have previously been shown to have increased circulating levels of endothelial- and leukocyte-derived MPs compared with nonFH patients (21). However, quantification of MPs in this study was achieved using flow cytometry, which is not considered optimal due to the technique’s lack of sensitivity for particles <200 nm. To our knowledge, no data exist detailing the effects of apheresis on MPs in individuals with FH, though other extracorporeal methods have been previously shown to remove MPs (22, 23).

Here we aimed to characterize size, concentration, cellular origin, fatty acids, and thrombin generation of MPs in FH patients undergoing apheresis, hypothesizing that this treatment would reduce circulating MPs as well as LDL.

## METHODS

### Patients

Twelve patients with clinically significant dyslipidemia undergoing fortnightly apheresis consented to take part in the study. For clinical reasons patients underwent treatment using three different techniques: polyacrylate whole blood adsorption (DALI® n = 8), whole blood dextran sulfate adsorption (n = 1), or plasma dextran sulfate adsorption (n = 3), as described previously (24). Patients attended the Lipid Unit at University Hospital Llandough, Cardiff for apheresis treatment as part of their normal clinical care. Patients fasted for at least 4 h prior to attendance and took their prescribed medication for at least 1 h prior to the study, excluding vasoactive medications from which patients were asked to refrain. Routine anthropometric measurements were carried out prior to apheresis treatment. After 15 min of rest, vascular access was gained using 16 gauge 25 mm fistula needles into two anatomically distinct upper limb veins or by arteriovenous fistula. Blood samples were then drawn sequentially prior to and immediately after completion of apheresis, approximately 3 h later. Seven healthy volunteers (free of CVD and medication) were also recruited to the study to compare MP concentration, size distribution, cellular origin, and fatty acid profile with FH individuals. Ethical approval for obtaining blood samples was provided by the South East Wales Research Ethics Committee.

### Biochemical measurements

Blood samples were collected as described above into EDTA and citrate vacutainers. An Architect automated analyzer (Abbott Diagnostics, Berkshire, UK) was used to measure total serum cholesterol (TC), HDL, and triglycerides; LDL was then estimated using the Friedewald equation. Glucose levels were determined by using the Architect chemistry system (Abbott Diagnostics) and high sensitivity C-reactive protein (hsCRP) was measured using nephelometry (BN™ II system, Dade Behring, UK). Blood pressure (BP) measurements were taken with patients seated using the Vicorder system (Skidmore Medical, UK) as part of a separate study.

### MP isolation

Blood, collected as above, was immediately centrifuged (1,509 *g*, 10 min, 4°C) to obtain platelet-poor plasma (PPP). EDTA plasma was snap-frozen in liquid nitrogen. Citrate plasma was ultracentrifuged (100,000 *g*, 1 h, 4°C) and the MP pellet was resuspended in either PBS and snap-frozen, in liquid nitrogen (for GC analysis), or in PBS containing 0.05% (v/v) Tween 20 (for MP size and concentration). The latter was then passed through a 1 μm filter (Supelco, Sigma-Aldrich, UK) and slow-frozen overnight at −80°C in a Mr Frosty (Nalgene, Thermo Scientific, UK). Plasma and MPs were maintained at −80°C until analysis.

### MP size and concentration

Two techniques were used to measure MP size and concentration: tunable resistive pulse sensing (TRPS) and nanoparticle tracking analysis (NTA). TRPS was carried out using the qNano (Izon Science, New Zealand), which uses an electrophoresis-based method to determine the size and concentration of MPs on a particle-by-particle basis. Particles pass through a tunable nanopore which detects particles within a specific size range. Therefore, in order to analyze MP size and concentration across a full spectrum, nanopore 100 (np100) and nanopore 200 (np200) were used. NTA was undertaken using the NanoSight LM10 (NanoSight Ltd., UK) with settings as previously described (25) using the NTA software (version 2.3). NTA uses the light scattering by MPs and tracks their Brownian motion in suspension over time to relate this to MP size and concentration. Sixty second videos were recorded in replicates of five per sample with camera sensitivity and detection threshold set to 14–16 and 5–6, respectively.

### MP origin

Flow cytometry was used to determine MP origin using a custom-built BD FACSAria II (BD Biosciences, CA). Forward scatter area (FSC-A) and side scatter area (SSC-A) were set to log scale and MPs were gated based on their FSC-A/SSC-A profile and in relation to platelets in fresh plasma. MP pellets were resuspended in 1× 0.22 μm-filtered annexin V binding buffer (BD Biosciences) and 100 μl of this was used for staining. MPs were stained in the dark (15 min, room temperature) with annexin V-FITC (1.57 μg/ml), CD41-PE-Cy5 (0.12 μg/ml), CD11b-PE-Cy7 (7.9 μg/ml), CD144-APC (4.1 μg/ml), and CD235a-PB (7.7 μg/ml) as markers of MPs, platelets, monocytes, endothelial cells, and erythrocytes, respectively (all antibodies from BioLegend, CA). Data were exported from the FACSDiva™ software (version 6) and subsequently analyzed in FlowJo software (version 9.6.4; Tree Star Inc., OR).

### MP fatty acid profile

Fatty acid profiles were analyzed using GC-flame ionization detection (FID) as described previously (26). Briefly, lipids were extracted from 200 μl of plasma or isolated MP samples (250 μl) with chloroform:methanol (1:2, v/v) by the method of Garbus et al. (27). Fatty acid methyl esters (FAMEs) were prepared by incubation for 2 h with 2.5% H_2_SO_4_ in dry methanol:toluene (2:1, v/v) at 70°C. A known amount of C19:0 (nonadecylic acid; Nu-Chek Prep, Inc., Elysian, MN) was added as an internal standard, so that subsequent quantification of peaks (and, consequently, lipids) could be performed. FAMEs were analyzed by GC using a Clarus 500 gas chromatograph (Perkin-Elmer 8500, Norwalk, CT), fitted with a 30 m × 0.25 mm inner diameter, 0.25 μm film thickness capillary column (Elite 225, Perkin-Elmer). The column temperature was held at 170°C for 3 min, and then temperature-programmed to 220°C at 4°C/min. Nitrogen was the carrier gas at a flow rate 2 ml/min. FAMEs were identified routinely by comparing retention times of peaks with those of standard (Supelco 37 Component FAME Mix, Sigma-Aldrich and Nu-Chek Prep, Inc.). Perkin-Elmer Total Chrom Navigator software was used for data acquisition.

### MP thrombin generation

To provide a working reservoir of plasma in which to test thrombin generation of MP, blood was drawn gently from healthy volunteers into a syringe containing 6 mM trisodium citrate (Sigma-Aldrich) and 20 μg/ml corn trypsin inhibitor (Cambridge BioScience, UK) and centrifuged (1,024 *g*, 10 min, 4°C) to yield “vehicle” PPP. Samples were then stored at −80°C until analysis.

To asses MP thrombin generation, calibrated automated thrombography was used, as described previously (28), with minor modifications. Samples were measured in duplicate using 96-well plates (round-bottomed, Immulon 2HB, Thermo Scientific). Eighty microliters of vehicle PPP (containing endogenous clotting factors) were added to each well with 20 μl of diluted HEPES/NaCl buffer (pH 7.4) tissue factor (TF) solution to yield a final concentration of 1 pM (Innovin, Sysmex UK Ltd., UK). FH MP samples were assayed for thrombin generation both with and without exogenous TF addition. Therefore, MPs (20 μl) were added to sample wells with the addition of either saline (20 μl, 0.9% NaCl) or TF (20 μl, 1 pM final). Each sample was calibrated to a well containing 80 μl of PPP and 40 μl of thrombin calibrator (600 nM, Synapse BV, The Netherlands). The plate was then warmed to 37°C for 5 min before addition of fluorogenic substrate (20 μl, Z-Gly-Gly-Arg-AMC, Bachem, UK). The fluorescent signal was then measured using a Fluoroskan Ascent plate reader (ThermoLabsystems, Finland) equipped with a 390/460 nm filter set (excitation/emission) at 15 s intervals until the thrombin generation reaction was complete. Data were analyzed using Thrombinoscope™ software (Synapse BV, The Netherlands) and correlated with MP concentration data.

### Statistical analysis

Data are presented as mean ± SEM. A paired *t*-test (two-tailed) or a Wilcoxon matched pairs test was used for parametric and nonparametric data, respectively. Analyses were conducted using GraphPad Prism (version 6; GraphPad Software Inc., CA) and *P* < 0.05 was considered statistically significant.

## RESULTS

### Anthropometric and biochemical data

Of the 12 participants in the study, 9 were male and 3 female with a mean age of 57.9 ± 10.3 years and a mean BMI of 30.0 ± 4.0 kg/m^2^. Biochemical measurements are summarized in **Table 1**. Apheresis reduced TC, triglycerides, HDL, LDL hsCRP, and systolic BP. No changes were observed in glucose levels, diastolic BP, or heart rate.

**TABLE 1.** Biochemical and physiological measurements pre- and post-apheresis

	Pre-apheresis	Post-apheresis	Change (%)	*P*
TC (mmol/l)	6.1 ± 0.5	2.7 ± 0.2	−57.1	<0.0001*^a^*
HDL (mmol/l)	1.1 (0.4–2.3)	0.9 (0.2–2.1)	−21.0	0.003*^a^*
Triglycerides (mmol/l)	1.8 ± 0.2	0.9 ± 0.1	−50.0	<0.0001*^a^*
LDL (mmol/l)	4.1 ± 0.4	1.4 ± 0.2	−66.1	<0.0001*^a^*
TC/HDL	5.8 (3.3–10.0)	3.1 (1.9–8.2)	−41.9	0.0005*^a^*
Glucose (mmol/l)	5.7 ± 0.3	6.1 ± 0.3	8.4	0.07
hsCRP (mg/l)	0.8 (0.2–16.9)	0.6 (0.2–13.8)	−29.8	0.003*^a^*
Systolic BP (mmHg)	140 ± 5	148 ± 6	6.5	0.02*^a^*
Diastolic BP (mmHg)	81.8 ± 2.8	82.8 ± 2.7	1.4	0.45
Heart rate (bpm)	55.8 ± 2.9	58.8 ± 3.4	5.8	0.09

Data are presented as mean ± SEM or median (range), n = 12. bpm, beats per minute.

a Denotes statistical significance

### MP size and concentration pre- versus post-apheresis

**Fig. 1.**
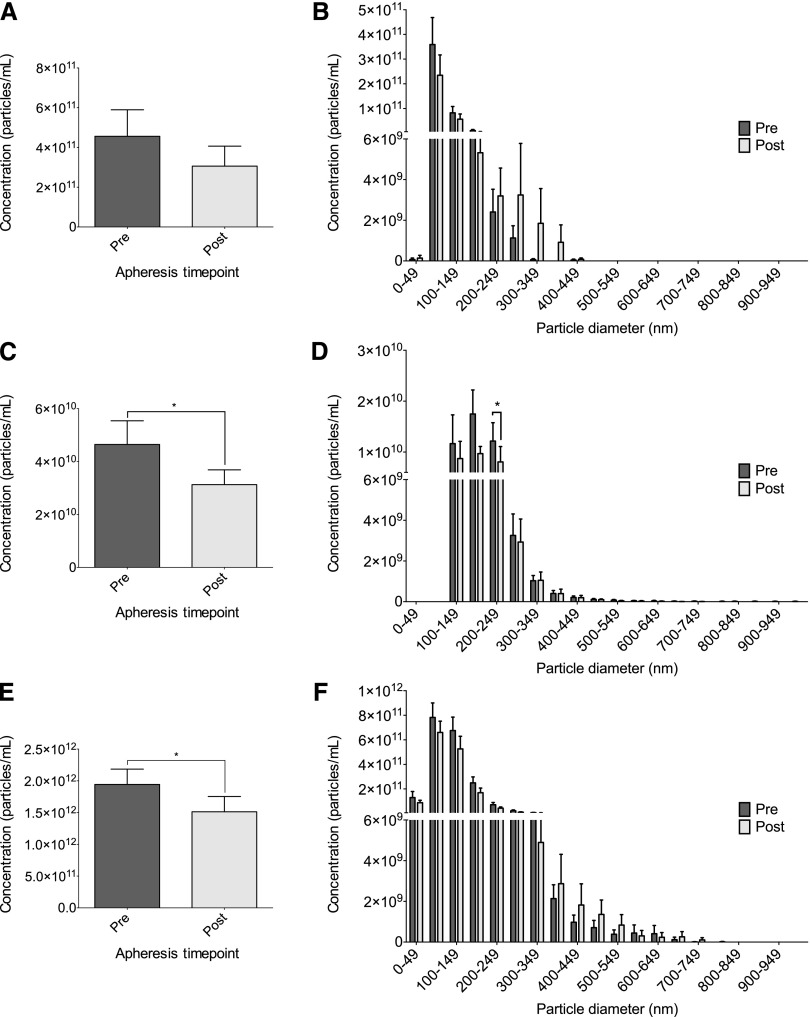
MP concentration and size distributions pre- and post-apheresis. MP size and concentration were measured in pre- and post-apheresis samples using TRPS (np100 and np200) and NTA. Mean concentration of MPs pre- and post-apheresis is shown for TRPS np100 (A), TRPS np200 (C), and NTA (E). Size/concentration distribution of MPs pre- and post-apheresis is shown for TRPS np100 (B), TRPS np200 (D), and NTA (F). Concentrations are given in particles/ml of plasma. Data are presented as mean ± SEM (n = 12). *P < 0.05.

Two techniques, TRPS (using np100 and np200) and NTA, were used to analyze MP size and concentration; therefore, the detectability was compared for each technique pre- and post-apheresis (supplementary Fig. IA, B). The size distribution of MPs was similar for TRPS (np100 and np200) and NTA, though the measured concentration varied greatly between the two techniques. Mode size of MPs did not change following apheresis by any technique [81.1 ± 19.6 nm to 78.4 ± 16.7 nm, *P* = 0.3 for TRPS (np100); 170.3 ± 40.6 nm to 163.6 ± 29.2 nm, *P* = 0.18 for TRPS (np200); and 93.3 ± 21 nm to 88.2 ± 14.7 nm, *P* = 0.32 for NTA]. TRPS (np100) measured no difference in concentration pre- to post-apheresis (4.6 × 10^11^ particles/ml ± 1.3 × 10^11^ particles/ml to 3.1 × 10^11^ particles/ml ± 1.0 × 10^11^ particles/ml, *P* = 0.18; **Fig. 1A**). However, TRPS (np200) and NTA both measured a decrease in MPs pre- versus post-apheresis [4.7 × 10^10^ particles/ml ± 8.8 × 10^9^ particles/ml to 3.1 × 10^10^ particles/ml ± 5.6 × 10^9^ particles/ml and 1.9 × 10^12^ particles/ml ± 2.4 × 10^11^ particles/ml to 1.5 × 10^12^ particles/ml ± 2.4 × 10^11^ particles/ml, *P* = 0.013 and *P* = 0.025; Fig. 1C and Fig. 1E for TRPS (np200) and NTA, respectively]. Total cholesterol was measured in the MP fraction and was below the detectability of the assay (<0.01 mmol/l). Size/concentration distributions of MPs are shown pre- to post-apheresis for each technique. TRPS (np100) and NTA show no preferential reduction according to MP size (Fig. 1B and Fig. 1F, respectively), whereas TRPS (np200) shows a reduction in MPs between 200 and 249 nm (Fig. 1D, *P* = 0.01). The type of apheresis received by patients was not shown to affect MP concentration (supplementary Table I); however, the study was not powered to measure this as an endpoint. Comparison of MPs in FH patients with those in healthy volunteers showed a trend (nonsignificant) toward an increase in total concentration; however, the size distribution showed individuals with FH had increased concentrations of MPs between 50 and 100 nm (*P* < 0.05, supplementary Fig. IIA and supplementary Fig. IIB, respectively).

**TABLE 2.** Fatty acid composition of the plasma and MP fraction

	Pre-apheresis			Post-apheresis		
Fatty Acid	Plasma Composition (%)	MP Composition (%)	*P*	Plasma Composition (%)	MP Composition (%)	*P*
C14:0	0.6 ± 0.1	1.09 ± 0.1	0.04*^a^*	0.9 ± 0.1	0.6 ± 0.07	0.008*^a^*
C14:1	0.04 ± 0.01	0.1 ± 0.02	0.006*^a^*	0.06 ± 0.1	0.1 ± 0.08	0.4
C16:0	15.7 ± 3.2	27.3 ± 3.2	0.07	10.5 ± 3.9	25.1 ± 1.2	0.002*^a^*
C16:1n7	2.5 ± 0.6	3.4 ± 0.4	0.4	2.0 ± 0.8	4.6 ± 0.7	0.02*^a^*
C18:0	5.0 ± 0.8	8.2 ± 0.9	0.07	3.1 ± 1.3	10.6 ± 0.6	<0.001*^a^*
C18:1n9	15.0 ± 4.2	6.2 ± 3.3	0.04*^a^*	10.1 ± 6.0	17.7 ± 5.4	0.4
C18:1n7	11.3 ± 4.2	32.5 ± 3.4	0.003*^a^*	31.8 ± 7.0	17.4 ± 5.4	0.1
C18:2n6	22.2 ± 5.8	13.3 ± 1.7	0.03*^a^*	14.0 ± 3.6	15.3 ± 1.2	0.7
C20:1	0.4 ± 0.1	0.3 ± 0.03	0.6	0.4 ± 0.06	0.4 ± 0.07	0.3
C20:2n6	1.7 ± 1.0	0.6 ± 0.09	0.2	1.5 ± 0.1	0.8 ± 0.09	<0.001*^a^*
C20:5n3	0.9 ± 0.2	0.7 ± 0.15	0.03*^a^*	2.2 ± 0.4	0.9 ± 0.15	0.002*^a^*
C22:0	0.3 ± 0.05	0.14 ± 0.06	0.02^a^	0.5 ± 0.07	0.14 ± 0.04	0.001*^a^*
C22:3n3	0.1 ± 0.03	0.15 ± 0.09	0.7	0.2 ± 0.04	0.02 ± 0.01	<0.001*^a^*
C22:3n6	0.4 ± 0.1	0.2 ± 0.1	0.09	0.9 ± 0.4	0.03 ± 0.03	0.004*^a^*
C22:5n3	0.2 ± 0.06	0.07 ± 0.04	0.04*^a^*	0.2 ± 0.1	0.3 ± 0.3	0.8
C22:6n3	1.2 ± 0.2	1.1 ± 0.2	0.09	2.4 ± 0.6	1.2 ± 0.2	0.09
C24:0	0.07 ± 0.03	0.002 ± 0.002	0.009*^a^*	0.2 ± 0.05	0.02 ± 0.02	0.003*^a^*
C24:1n9	0.9 ± 0.2	0.2 ± 0.1	0.006*^a^*	1.6 ± 0.7	0.04 ± 0.02	0.08

Individual fatty acid composition of the plasma and MPs were directly compared in pre-apheresis and post-apheresis samples. Data are presented as mean ± SEM (n = 12).

a Denotes statistical significance.

### Effect of apheresis on MP origin

**Fig. 2.**
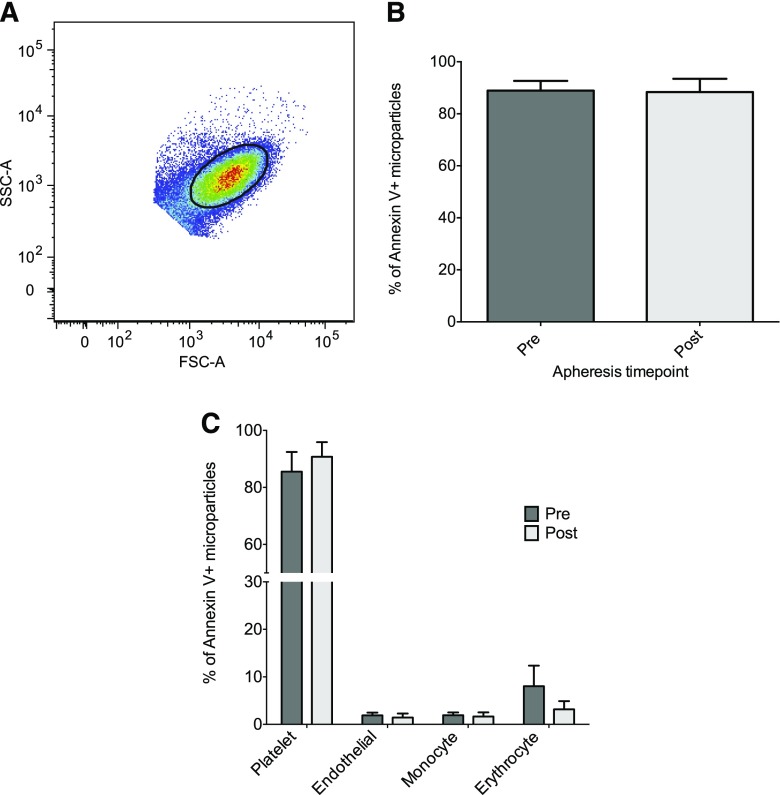
MP origin following apheresis. MPs from pre- and post-apheresis samples were analyzed by flow cytometry to determine cellular origin. FSC-A and SSC-A of platelets from fresh plasma were used to determine a submicron gate where only annexin V-positive MPs were analyzed. A: A representative dot blot of FSC-A versus SSC-A indicates the position of the MP gate (kept consistent for all samples). Samples were stained with annexin V, CD41, CD144, CD235a, and CD11b (B) to identify the proportion derived from platelets, endothelial cells, erythrocytes, and monocytes, respectively (C). Data are presented as mean ± SEM (n = 12).

MPs falling within the MP gate (**Fig. 2A**) showed no change in apportion of annexin V positivity following apheresis (Fig. 2B). Of these annexin V-positive MPs, there were also no changes in the proportions derived from platelets (CD41), endothelial cells (CD144), monocytes (CD11b), or erythrocytes (CD235a) (Fig. 2C). MPs positive for both annexin V and CD41 accounted for the majority (∼90%) of MPs measured. The same was also true for MPs in healthy volunteers, though there was a minor increase in the proportion of endothelial-derived MPs in individuals with FH compared with healthy volunteers (*P* = 0.03, supplementary Fig. III). The apportion of MPs showed no observable differences depending on the type of apheresis (supplementary Table I), though the study was not powered on this basis.

### MP fatty acid concentration and profile pre- versus post-apheresis

**Fig. 3.**
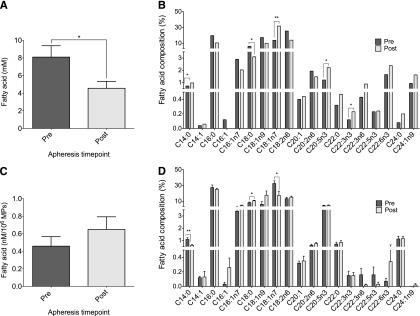
GC-FID analysis of MPs following apheresis. Total fatty acid concentration of plasma and MPs (A and C, respectively) followed by fatty acid profiling to determine compositional changes pre- to post-apheresis (B and D, respectively). Data are presented as mean ± SEM (n = 12). *P < 0.05, **P < 0.01.

Little is known about the lipid concentration of MPs, thus we sought to determine the fatty acid concentration and profile of MPs compared with that of the corresponding plasma and to observe the effect of apheresis. Total plasma fatty acid concentration decreased following apheresis (8.1 ± 1.3 mM to 4.6 ± 0.8 mM; *P* = 0.01), though this was not mirrored in the MP fraction (**Fig. 3A** and Fig. 3C, respectively). Five individual fatty acids were altered in the plasma following apheresis (*P* < 0.05): C14:0 (myristic acid), C18:0 (steric acid), C18:1n7 (*cis*-vaccenic acid), C20:5n3 (eicosapentaenoic acid), and C22:3n3 (docosatrienoic acid); the former three also being altered in the MP fraction (*P* < 0.05, Fig. 3B and Fig. 3D, respectively). Interestingly, comparison of the compartments, plasma and MPs, revealed 10 fatty acids differed in composition (*P* < 0.05) (**Table 2**). This was true in pre- and post-apheresis samples; however, the 10 fatty acids were not the same. Comparing FH patients to healthy volunteers, both plasma and MP fatty acid concentration were elevated in individuals with FH (*P* = 0.02 and *P* = 0.01, supplementary Fig. IVA and supplementary Fig. IVC, respectively). Eight fatty acids were different in plasma and nine were different in MPs comparing healthy volunteers and individuals with FH (*P* < 0.05, supplementary Fig. IVB and supplementary Fig. IVD, respectively), and apheresis had a similar effect on fatty acids in either compartment. This was consistent with all types of apheresis received (supplementary Table I).

### MP thrombin generation

Thrombin generation of MPs was analyzed pre- to post-apheresis and was also correlated with total MP concentrations. No change was observed in MP peak thrombin generation pre- to post-apheresis (14.8 ± 2.9 nM to 16.6 ± 4.1 nM, *P* = 0.6). However, MP thrombin generation over time [area under the curve (AUC)] was positively correlated with MP concentration measured by TRPS (np100) and NTA (*r* = 0.626, *P* = 0.001 and *r* = 0.424, *P* = 0.04, respectively). Thrombin AUC showed no correlation with MP concentration measured by TRPS (np200). Furthermore, when TF was added to MPs (to exogenously initiate maximum thrombin generation), thrombin AUC no longer correlated with MP concentration.

## DISCUSSION

Circulating MPs are reportedly elevated in a number of diseases, including in patients with severe hypercholesterolemia (21, 29). The present study shows novel data regarding the effects of apheresis on MP size, concentration, origin, fatty acid concentration, and thrombin generation in patients with FH. Our data demonstrate that apheresis reduces circulating MP concentration, the majority of which are annexin V-positive MPs derived from platelets.

Several methods exist to measure MPs, though often the technique employed is heavily dictated by the research question and protocol, and each exhibits unique advantages/limitations. Thus, we sought to employ two well-established methods for MP measurement in order to capture the full spectrum of MP sizes. To our knowledge, TRPS and NTA have not previously been subjected to a direct comparison in biological samples. Our data illustrate that the range of detectability of TRPS (np100 and np200) and NTA are similar, and although they differ vastly in reported MP concentration, taken together there is considerable agreement.

No change was observed with TRPS or NTA in mode particle size pre- versus post-apheresis. TRPS using the np200 and NTA showed a fall in MP concentration pre- versus post-apheresis. MPs within the range of 200–250 nm were reduced the most pre- versus post-apheresis, which is much greater than the size of LDL particles (30), indicating that the techniques are measuring a reduction in MPs and not LDL. MP concentration did not fall when measured with TRPS using the np100. This further supports that the observed fall in MP concentration by the np200 and NTA was not due to either technique detecting LDL, as the particulate size of LDL lies within the sensitivity of the np100 pore range. MPs were also shown to be elevated in the exosomal range in individuals with FH compared with healthy volunteers. Total MP concentration also appears elevated in individuals with FH, though this did not quite reach significance.

Flow cytometric measurement of MPs revealed no changes in annexin V positivity or cellular origin following apheresis. In keeping with previous data (21, 31, 32), platelet-MP occupied the majority of the MP population (88.9 ± 13%). Taken together with the fall in MP concentration, this would suggest that apheresis nonselectively removes MPs, the majority of which are annexin V positive and derived from platelets. These MPs have not only been shown to be elevated in a variety of disease states (33–38), but have also been shown to promote coagulation (19) and atherosclerotic plaque formation (39), and to be associated with atherothrombotic events (35). Nonselective removal of these MPs by apheresis may reduce the risk of thrombus formation by slowing the progression of atherosclerotic lesions, thereby complementing the effect of LDL removal. MPs in healthy volunteers were also found to be mainly annexin V positive and of platelet origin. It would appear that individuals with FH have a greater number of these circulating annexin V/platelet-positive MPs. Individuals with FH had a greater apportion of endothelial-derived MPs, perhaps suggesting a greater level of endothelial activation compared with healthy volunteers, although the percentage of the total was still low (<2%).

GC-FID was used to measure fatty acid concentration and composition in plasma and MPs. The relative atheroprotective mechanisms of unsaturated and polyunsaturated fatty acids are well-documented (40), as are the data implicating saturated fatty acids in arterial wall lipid accumulation and atherosclerotic plaque formation (41). MPs have been shown to carry a specific cargo of proteins, genetic material and small molecules, including fatty acids (42), that can initiate a pro-inflammatory response in target cells (20). Here, total fatty acid concentration of plasma was decreased following apheresis, however MP fatty acid levels did not change following apheresis. Thus, although the overall number of MPs decreases pre- versus post-apheresis, the fatty acid concentration per MP remains the same. Interestingly, apheresis seemed to affect individual fatty acids differently in plasma compared with the MP fraction; however, the physiological relevance of this remains to be elucidated. Furthermore, when plasma was directly compared with the MP fraction, the apportion of fatty acids was found to be different between compartments. This suggests that the fatty acid composition of MPs is independent to that of surrounding plasma, a concept we have previously found in a separate cohort of patients with polycystic ovary syndrome (unpublished observations). Several fatty acids were different in individuals with FH compared with healthy volunteers in both plasma and MPs, however, affected fatty acids and the trends (i.e., increase or decrease) were the same. Similarly to the findings in FH, in healthy volunteers the fatty acid composition of MPs did not reflect that of plasma.

The potential of MPs to generate thrombin was assessed using calibrated automated thrombography. No change was observed in MP peak thrombin generation pre- versus post-apheresis. However, total MP concentration measured by either TRPS (np100) or NTA showed a positive correlation with the total thrombin AUC, whereas MP concentration measured by TRPS (np200) showed no correlation. Taken together, this suggests a reduction in MPs is associated with decreased thrombin generation capacity and that smaller MPs, particularly exosomes, are associated with an increased total thrombin generation over time. We conclude this on the basis that both TRPS (np100) and NTA have an increased sensitivity for MPs in the exosomal range compared with TRPS (np200) (supplementary Fig. I). Furthermore, individuals with FH were shown to have an increased circulating population of smaller MPs compared with healthy individuals (supplementary Fig. II). Both TRPS (np100) and NTA showed a trend toward reduction in exosomal populations of MPs following apheresis (Fig. 1B and Fig. 1F, respectively), though this did not reach significance. This could suggest that the increased circulating population of exosomes in individuals with FH makes their MP fraction more procoagulant. Apheresis treatment nonselectively removes MPs and could potentially reduce the procoagulant potential of exosomes and smaller MPs. Our results confirm that MPs have endogenous TF activity and can stimulate thrombin generation, a finding in keeping with previous research (43, 44). When exogenous TF was added to MPs to stimulate thrombin generation, the correlation between MP concentration and AUC was lost, reflecting maximum thrombin generation.

Patients in this study received apheresis based on individual clinical requirements resulting in the use of three different types of apheresis treatment. Though there was no observable difference between apheresis technique and MP concentration (supplementary Table I), the current study was not designed to assess this. In vitro studies have shown the surface morphology of the adsorbent polymer may affect MP production (45), though this requires confirmation in vivo. FH patients were studied as part of their routine clinical outpatient treatment. Clearly, having now established that apheresis directly influences MP concentration, longitudinal studies will help to establish whether the reduction in atherogenic MPs is maintained while further exploring the physiological relevance this reduction in MPs has in regard to CVD pathology. The thrombin generation of patient MP samples was measured in the presence of pooled healthy plasma to specifically test the activity of MPs as opposed to whole patient plasma (that would likely reflect the total influence of apheresis). Future studies should assess the procoagulant activity of plasma pre- to post-apheresis to confirm this reduction in atherogenic MPs. Finally, annexin V binding was used to classify MP populations for identification of cellular origin using flow cytometry. It is acknowledged that many flow cytometers have a practical lower limit of around 200 nm. Therefore, smaller MPs, particularly exosomes, are below the detectability of these machines, and the fluorescence data obtained from a given sample does not completely reflect the full range of MP sizes observed by NTA and TRPS. Despite this, flow cytometry is the most reliable technique to assess surface antigen expression of MPs. Importantly, NTA and TRPS confirm that MPs within the range of 200–250 nm were reduced the most pre- versus post-apheresis, and are likely reflected by the flow cytometric results. The use of annexin V positivity to identify MP populations is used widely (31, 46, 47), but has recently been questioned (48). As the majority (∼90%) of MPs here were annexin V positive, we chose to accept this as our MP population for subsequent staining. Our rationale was based on the fact that despite not all MPs binding annexin V, only annexin V-positive MPs have been shown to possess procoagulant activity (48).

In summary, apheresis reduces the concentration of circulating MPs in patients with FH, the majority of which are annexin V and platelet positive. Though MP concentration is reduced, apheresis has no effect on the total fatty acid concentration of MPs. Fatty acid composition of MPs is unique and does not reflect that of the surrounding plasma. Each compartment is affected differently by apheresis, though the clinical relevance of this requires further investigation. MP concentration (particularly in the exosomal range) was found to positively correlate with total thrombin generation, suggesting that a reduction in MP concentration via apheresis in FH may reduce the ability of MPs to produce thrombin. The removal of MPs that are predominantly annexin V and platelet derived is a novel finding, supporting the notion that apheresis may have beneficial cardiovascular effects beyond lipoprotein removal. Future work should establish whether MP reduction during apheresis correlates with the longer-term benefits of this treatment.

## Supplementary Material

Supplemental Data
