# Supplementary material for: Lipoprotein-apheresis reduces circulating microparticles in individuals with familial hypercholesterolemia
Source: J Lipid Res. 2014 Oct;55(10):2064–72. doi: 10.1194/jlr.M049726 (PMC4173999; doi:10.1194/jlr.M049726)
Supplement: Supplemental Data [file supp_55_10_2064__index.html]

Lipoprotein-apheresis reduces circulating microparticles in individuals with familial hypercholesterolemia — Lipoprotein-apheresis reduces circulating microparticles in individuals with familial hypercholesterolemia — Supplemental Data 

# Lipoprotein-apheresis reduces circulating microparticles in individuals with familial hypercholesterolemia

## Supplemental Data

**Files in this Data Supplement:**

- Figure SI, Figure SII, Figure SIII, Figure SIV, Table SI - Size distributions of the techniques used for MP measurement. MP concentration and size distribution of healthy volunteers and FH. MP origin of healthy volunteers and FH. Plasma and MP fatty acid concentration and profile of healthy volunteers and FH. The effect of the type of apheresis on each MP parameter analysed.
