# Supplementary material for: Lipoprotein-apheresis reduces circulating microparticles in individuals with familial hypercholesterolemia
Source: J Lipid Res. 2014 Oct;55(10):2064–72. doi: 10.1194/jlr.M049726 (PMC4173999; doi:10.1194/jlr.M049726)
Supplement: Supplemental Data [file supp_M049726_jlr.M049726-1.pdf]

Supplemental data

Figure SI: Size distributions of the techniques used for MP measurement. A comparison of the spectrum of MPs measured by TRPS (np100 and np200) and NTA pre- (**A**) and post-apheresis (**B**). Concentration is given as particles per mL of plasma.

Figure SII: MP concentration and size distribution of healthy volunteers and FH. Comparison of the total MP concentration (**A**) and the size/concentration distribution (**B**) of MPs in healthy volunteers compared to individuals with FH. Measured using NTA, *p<0.05. Concentration is given as particles/per mL of plasma.

Figure SIII: MP origin of healthy volunteers and FH. Comparison of the MP Annexin V positivity (**A**) and cellular origin (**B**) of MPs from healthy volunteers compared to individuals with FH. *p<0.05

Figure SIV: Plasma and MP fatty acid concentration and profile of healthy volunteers and FH. Total fatty acid concentration of plasma (**A**) and MPs (**C)** was compared between healthy volunteers and individuals with FH. Individual fatty acid profiles of plasma (**B**) and MPs (**D**) were also compared between groups. *p<0.05, **p<0.01, ***p<0.001, ****p<0.0001.

Table SI: The effect of the type of apheresis on each MP parameter analysed. Breakdown of the effect of each type of apheresis used in the study on MP parameters analysed. DALI®; polyacrylate whole blood adsorption, n=8, PDSA; plasma dextran sulphate adsorption, n=3, and WBDSA; whole blood dextran sulphate adsorption, n=1. MP concentration pre- to post-apheresis is shown for both TRPS (np100 and np200) and NTA in particles per mL of plasma.

FIGURE SI

A

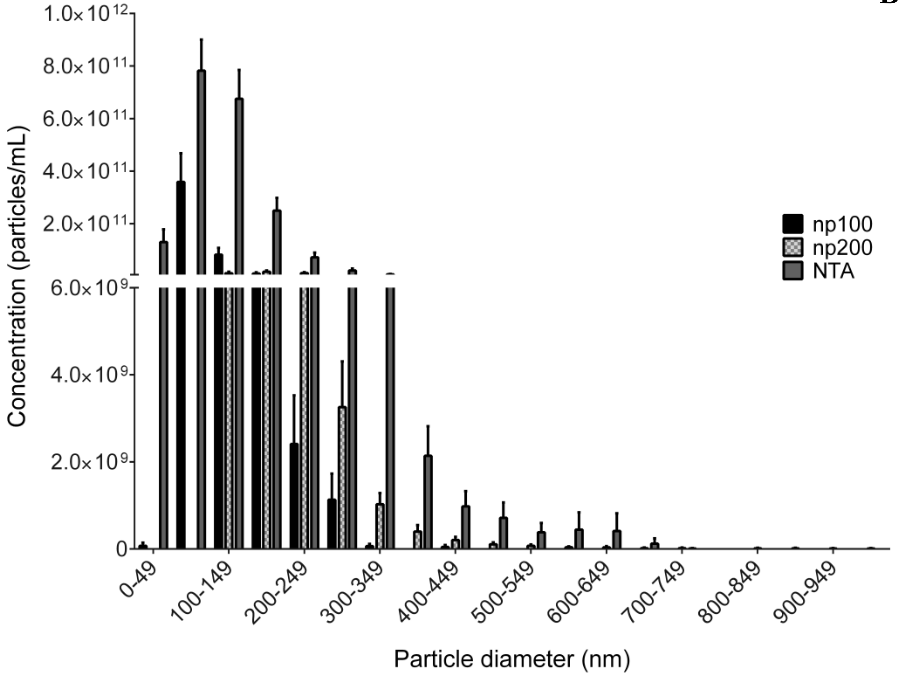

1.0×10^12
8.0×10^11
6.0×10^11
4.0×10^11
2.0×10^11
6.0×10^9
4.0×10^9
2.0×10^9
0
Concentration (particles/mL)
0-49
100-149
200-249
300-349
400-449
500-549
600-649
700-749
800-849
900-949
Particle diameter (nm)
np100
np200
NTA

B

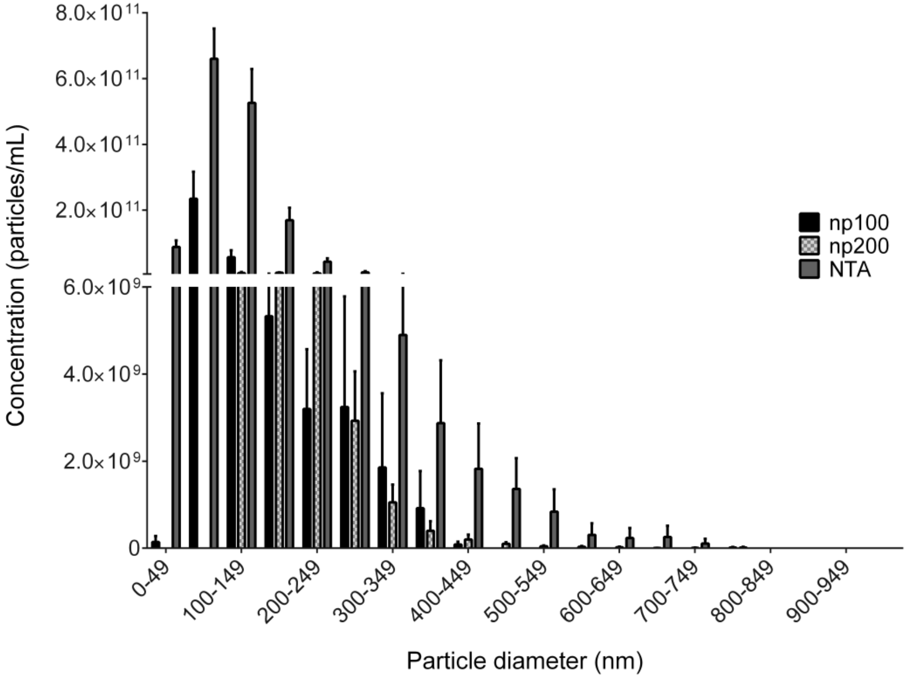

8.0×10^11
6.0×10^11
4.0×10^11
2.0×10^11
6.0×10^9
4.0×10^9
2.0×10^9
0
Concentration (particles/mL)
0-49
100-149
200-249
300-349
400-449
500-549
600-649
700-749
800-849
900-949
Particle diameter (nm)
np100
np200
NTA

FIGURE SII

A

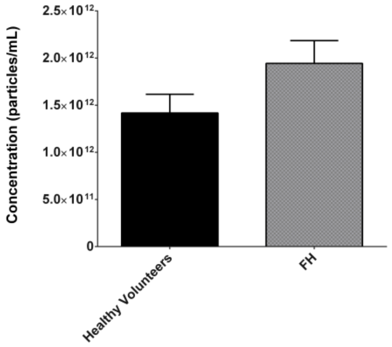

B

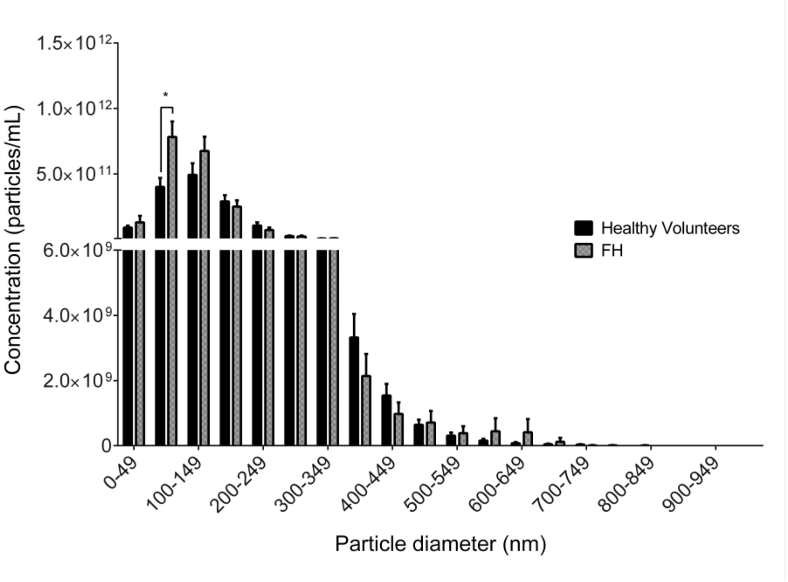

FIGURE SIII

A

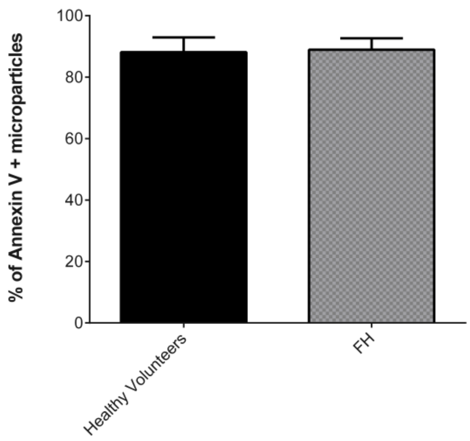

B

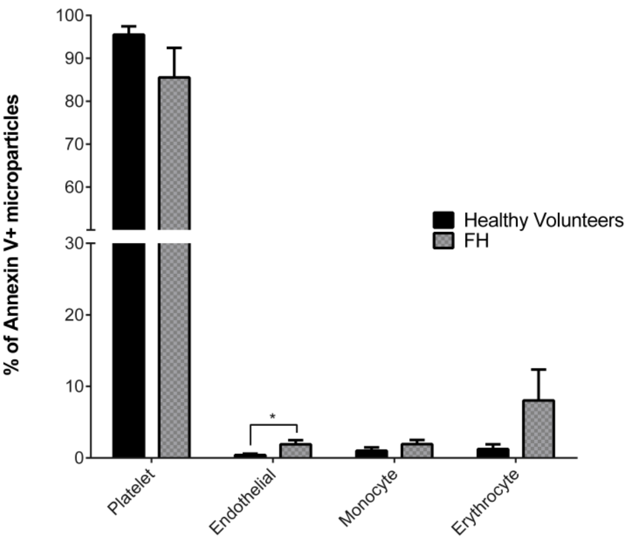

FIGURE SIV

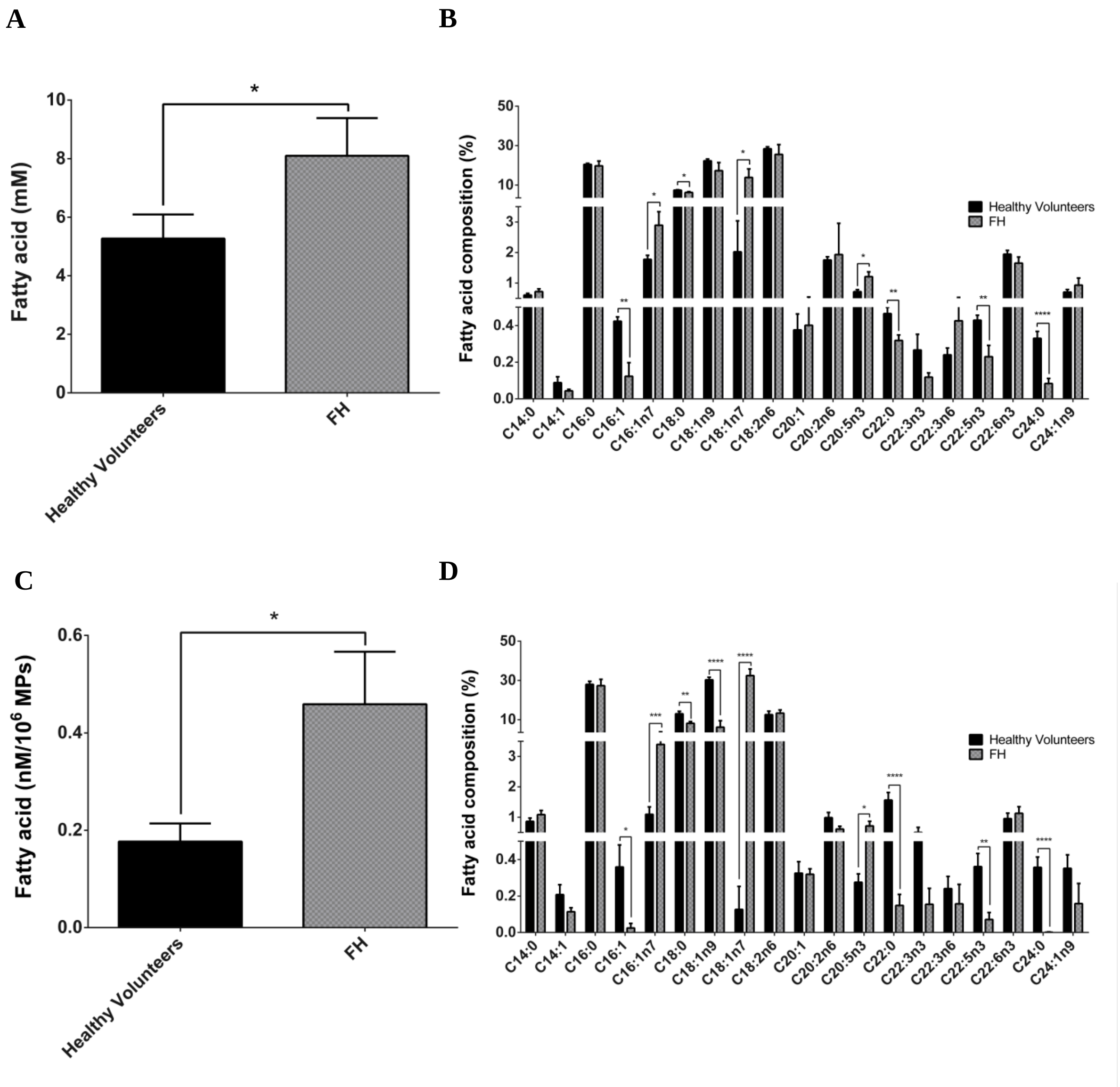
A
B
C
D
Fatty acid (mM)
Fatty acid (nM/10^6 MPs)
Healthy Volunteers
FH
Fatty acid composition (%)
Healthy Volunteers
FH
C14:0
C14:1
C16:0
C16:1
C16:1n7
C18:0
C18:1n9
C18:1n7
C18:2n6
C20:1
C20:2n6
C20:5n3
C22:0
C22:3n3
C22:3n6
C22:5n3
C22:6n3
C24:0
C24:1n9

TABLE SI

	TYPE OF APHERESIS		
MP MEASUREMENT	**DALI® (n=8)**	**PDSA (n=3)**	**WBDSA (n=1)**
**TRPS (np100)**<br>(particles/mL)	$3.6 \times 10^{11} \pm 1.2 \times 10^{11}$ to $3.3 \times 10^{11} \pm 1.3 \times 10^{11}$	$5.0 \times 10^{11} \pm 4.1 \times 10^{11}$ to $3.0 \times 10^{11} \pm 2.6 \times 10^{11}$	1.1×10^{11} to 1.3×10^{11}
**TRPS (np200)**<br>(particles/mL)	$4.9 \times 10^{10} \pm 1.1 \times 10^{10}$ to $3.6 \times 10^{10} \pm 7.0 \times 10^{9}$	$3.7 \times 10^{10} \pm 2.2 \times 10^{10}$ to $2.1 \times 10^{10} \pm 1.1 \times 10^{10}$	5.3×10^{10} to 2.3×10^{10}
**NTA**<br>(particles/mL)	$1.8 \times 10^{12} \pm 2.6 \times 10^{11}$ to $1.6 \times 10^{12} \pm 2.8 \times 10^{11}$	$2.1 \times 10^{12} \pm 7.7 \times 10^{11}$ to $1.3 \times 10^{12} \pm 7.0 \times 10^{11}$	$2.4 \times 10^{12} \pm$ to 1.7×10^{12}
**Annexin V positivity**<br>(%)	91.6 ± 3.4 to 93.0 ± 3.6	79.4 ± 11.8 to 75.6 ± 18.5	95.6 to 89.9
**CD41 positivity**<br>(% of Annexin V +)	91.3 ± 4.0 to 94.7 ± 2.9	66.0 ± 25.2 to 79.1 ± 20.6	89.9 to 93.2
**CD144 positivity**<br>(% of Annexin V +)	1.8 ± 0.8 to 0.6 ± 0.2	2.7 ± 1.2 to 3.7 ± 3.5	0.6 to 1.2
**CD11b positivity**<br>(% of Annexin V +)	1.8 ± 0.8 to 0.9 ± 0.4	2.6 ± 1.2 to 3.6 ± 3.5	0.8 to 1.2
**CD235a positivity**<br>(% of Annexin V +)	4.7 ± 2.4 to 1.6 ± 0.5	19.3 ± 16.4 to 7.3 ± 7.1	1.0 to 3.5
**Plasma fatty acid**<br>(mM)	8.7 ± 1.9 to 4.9 ± 1.1	7.8 ± 1.4 to 3.1 ± 0.6	4.4 to 6.1
**MP fatty acid**<br>(nM/10^6 MPs)	0.4 ± 0.1 to 0.5 ± 0.2	0.6 ± 0.3 to 1.1 ± 0.3	0.3 to 0.3
